# Pulmonary veno-occlusive disease as a cause of severe pulmonary hypertension in a dog

**DOI:** 10.1186/s13028-018-0433-1

**Published:** 2018-12-05

**Authors:** Marjolein Lisette den Toom, Guy Grinwis, Robert-Jan van Suylen, Susanne Adetokunbo Boroffka, Pim de Jong, Frank Geurt van Steenbeek, Viktor Szatmári

**Affiliations:** 1 Department of Clinical Sciences of Companion Animals, Faculty of Veterinary Medicine, Utrecht University, Yalelaan 108, 3508 TD Utrecht, The Netherlands; 2 Department of Pathobiology, Faculty of Veterinary Medicine, Utrecht University, Yalelaan 1, 3485 CL Utrecht, The Netherlands; 3 Pathology-DNA, Location Jeroen Bosch Hospital, s’ Hertogenbosch, The Netherlands; 4 Division of Diagnostic Imaging, Department of Clinical Sciences of Companion Animals, Faculty of Veterinary Medicine, Utrecht University, Yalelaan 108, 3508 TD Utrecht, The Netherlands; 5 Department of Radiology, University Medical Centre Utrecht, Utrecht, The Netherlands

**Keywords:** Canine, Pulmonary arterial hypertension, Pulmonary capillary hemangiomatosis

## Abstract

**Background:**

Pulmonary veno-occlusive disease (PVOD) is a rare cause of pulmonary arterial hypertension (PAH) in humans and can be classified in idiopathic, heritable, drug and radiation-induced, and associated with connective tissue disease or human immunodeficiency virus infection. Recently, biallelic mutations of the *EIF2AK4* gene have been discovered as a cause for an autosomal recessive form of PVOD in humans. In dogs, PAH is poorly characterized and is generally considered to be idiopathic or secondary to (for example) congenital left-to right cardiovascular shunts or heartworm disease. However, recently, the pathologic features resembling human PVOD were retrospectively described in *post*-*mortem* lung samples of dogs presenting with respiratory distress and idiopathic pulmonary hypertension (PH), which suggests that PVOD contributes to an unknown percentage of cases with unexplained PH. In dogs, information on the clinical presentation of PVOD is scarce and the cause and pathogenesis of this disease is still unknown.

**Case presentation:**

An 11-year-old, intact male German Shepherd dog (GSD) was presented with a 2-day history of acute-onset dyspnea and generalized weakness. Physical examination, laboratory analysis, thoracic radiography, echocardiography, a computed tomography scan and an *ante mortem* lung biopsy demonstrated severe arterial hypoxemia and severe PH but were not diagnostic for a known disease syndrome. Based on the poor reaction to therapy with oxygen, sildenafil, pimobendan and dexamethasone the dog was euthanized. Histopathology of the lungs showed venous and arterial remodelling, segmental congestion of alveolar capillaries and foci of vascular changes similar to human pulmonary capillary hemangiomatosis, indicating that the dog suffered from PVOD. Whole genome sequencing analysis was performed on the case and a healthy GSD. Validation was performed by Sanger sequencing of five additional GSD's unknown for any form of respiratory stress and aged ≥ 10 years. No causal variants were found in the genes that are known to be involved in human PVOD and PAH.

**Conclusions:**

This case report confirms that PVOD should be a diagnostic consideration in dogs presenting with dyspnea and unexplained PH. In the present case, no casual genetic mutations known to be involved in humans with PVOD and PAH were found.

## Background

Pulmonary veno-occlusive disease (PVOD) is a rare cause of pulmonary hypertension (PH) in humans. It is characterized by preferential remodelling of the pulmonary venules and leads to a progressive increase in pulmonary vascular resistance, right heart failure and death [1]. PVOD is further classified in humans based on its aetiology and can be divided in the following forms: idiopathic, heritable, drug- and radiation-induced, and associated with connective tissue disease or human immunodeficiency virus infection [1]. Recently, biallelic mutations of the *eukaryotic translation initiation factor 2 alpha kinase (EIF2AK4)* gene have been discovered as a cause for an autosomal recessive form of PVOD in people [2]. Although PVOD is classified as belonging to the group of pulmonary arterial hypertension (PAH) in the current classification systems of PH [3], PVOD has been given a separate subgroup. In this subgroup PVOD is combined with pulmonary capillary haemangiomatosis (PCH), because PVOD and PCH are considered different expressions of the same disorder. PCH is characterised by exuberant proliferation of endothelial cells of the capillaries. Pathological studies of humans and dogs indicate marked overlap in the histological findings of PVOD and PCH. The reason for the implementation of this subgroup was to emphasize both similarities and important differences between PAH and PVOD. Although the clinical presentation is similar, PVOD typically is more aggressive and has a poorer prognosis. Furthermore, in contrast to patients with PAH, standard therapy with vasodilators can result in life-threatening pulmonary oedema in patients with PVOD [1, 4, 5].

Histopathological abnormalities are seen in all three compartments of the pulmonary microcirculation in PVOD, although there is a preferential involvement of the pulmonary venous system. Venular lesions include intimal fibrosis of small pre-septal venules. Capillary lesions are characterized by exuberant proliferation of endothelial cells (PCH). Arterial lesions resemble those of PAH with intimal fibrosis and medial hypertrophy, but complex plexiform lesions are absent [6]. Common radiographic findings in humans include septal lines and poorly defined centrilobular ground-glass opacities, although they are nonspecific. Lymphadenopathy, pleural effusions and enlarged pulmonary arteries have also been described [7].

In veterinary medicine, PH is a well-known disease in dogs. Most common reported causes of PH are PH secondary to left-sided heart disease, PH secondary to pulmonary disease and/or hypoxia, PH secondary to congenital left-to right cardiovascular shunts and PH secondary to infections with *Dirofilaria immitis* [8] or *Angiostrongylus vasorum* [9, 10]. PAH is poorly characterized and is generally considered to be idiopathic or secondary to congenital left-to right cardiovascular shunts, heartworm infections or necrotising vasculitis [8]. To the author’s knowledge, no association with drugs or toxins, connective tissue diseases or heritability of PAH has been described in dogs and PVOD was not recognized as a separate subtype of PAH until recently. In 2016, pathologic features resembling human PVOD were retrospectively diagnosed in 11 dogs with severe idiopathic PH and dyspnea by Williams et al. [11]. This group of dogs consisted of various breeds with a median age of 10.5 years and no sex predilection was observed. Information about clinical presentation and diagnostic findings were however limited in this study.

## Case presentation

An 11-year-old, intact male German Shepherd dog (GSD) was referred to the emergency service of the Department of Clinical Sciences of Companion Animals of the Faculty of Veterinary Medicine of Utrecht University with a 2-day history of acute onset dyspnea and generalized weakness. Vaccination and deworming were performed regularly. The dog had visited Southern Europe 6 months before presentation. No drugs were administered prior to the development of the clinical signs and no environmental circumstances that could cause dyspnea (e.g. tobacco, organic solvent exposure, dust) were reported.

Physical examination showed a responsive but lethargic dog with generalized weakness, severe dyspnea, cyanotic mucous membranes, prolonged capillary refill time, weak peripheral pulses, tachycardia (heart rate 180 beats/min) and a grade one out of six systolic murmur with the point of maximal intensity over the right cardiac apex. Harsh lung sounds were heard on lung auscultation. Complete blood count (CBC) showed a mild mature leukocytosis (white blood cells: 18.9 × 10^9^/L; reference interval 4.5–14.6 × 10^9^/L) and a haematocrit of 56% (reference interval 42–61%). Biochemistry did not show any abnormalities. Arterial blood gas analysis showed a severe hypoxemia (PaO_2_: 48.5 mm Hg; reference interval 85–103 mm Hg) and mild hypocapnia (PaCO_2_: 27.0 mm Hg; reference interval: 32–43 mm Hg). The suspected cause for the hypocapnia was hyperventilation. D-dimer and antithrombin concentrations were within the reference intervals. *Dirofilaria immitis* antigen snap test (SNAP^®^ Heartworm RT Test, IDEXX Laboratories) and faecal examination (flotation and Baermann larval isolation technique) were both negative.

**Fig. 1:**
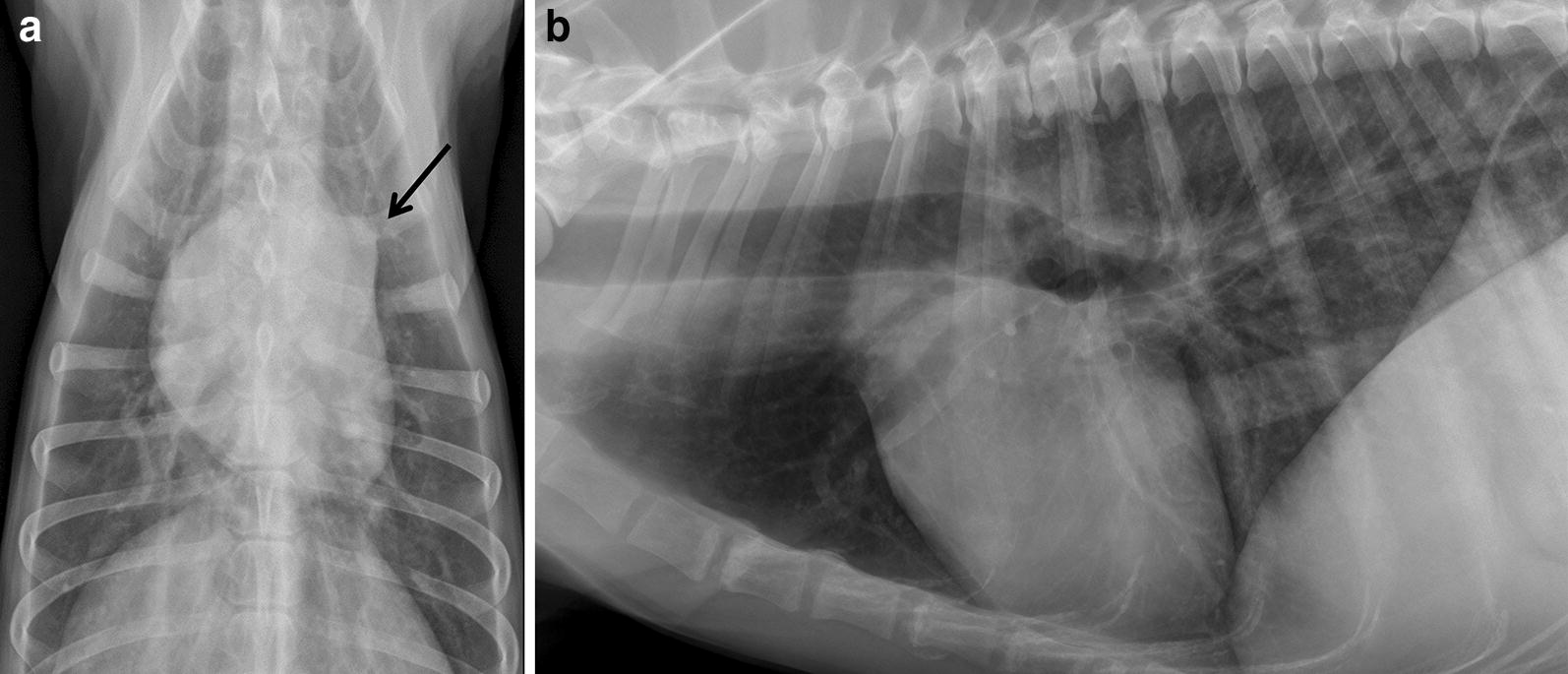
Dorso-ventral (**a**) and left lateral (**b**) thoracic radiographs. Thoracic radiographs demonstrating right-sided enlargement of the cardiac silhouette and a clear dilation of the pulmonary trunk on the dorso-ventral radiograph (arrow)

**Fig. 2:**
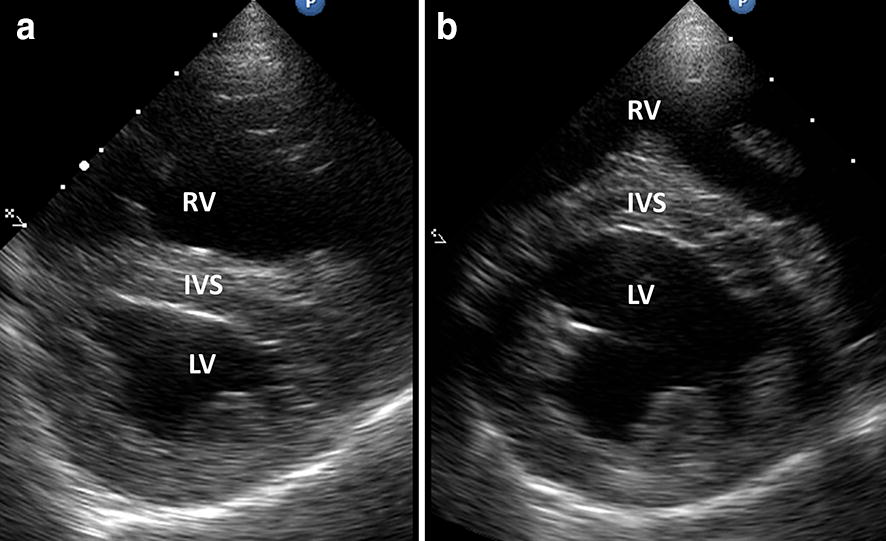
B-mode echocardiographic images from the right parasternal short axis view of the ventricles pre-and post-vasodilator therapy. **a** Echocardiogram at presentation: severe dilation of the right ventricle (RV), flattening of the interventricular septum (IVS) and a hypovolemic left ventricle (LV). **b** Echocardiogram 3 days after initiation of vasodilator therapy: almost complete normalization of cardiac dimensions and normal position of the IVS

Thoracic radiographs showed a dilation of the pulmonary artery trunk and right-sided cardiomegaly (Fig. 1). Echocardiography was severely complicated due to the severe anxiety and panting of the dog and was therefore limited. It showed severe right ventricular dilation, mild uniform dilation of the main pulmonary artery, systolic flattening of the interventricular septum and moderate tricuspid regurgitation (Fig. 2a). Application of the modified Bernoulli equation to the velocity of the tricuspid regurgitation jet showed an estimated systolic pulmonary artery pressure of 77 mm Hg, graded as severe PH (reference < 25 mm, severe > 75 mm Hg) [8]. The left ventricular dimensions were markedly reduced, consistent with left-sided volume depletion. A saline contrast echocardiography was performed, which was negative, thereby excluding intra- and extra-cardiac right-to-left shunting. To address the severe hypoxemia and PH the dog was placed in an oxygen cage with an inspired concentration of oxygen between 40 and 50%. Furthermore, 1.5 mg/kg/8 h oral sildenafil (Viagra^®^, Pfizer, New York, USA) and 0.25 mg/kg/12 h oral pimobendan (Vetmedin^®^, Boehringer Ingelheim, Germany) were administered. This therapy did not significantly affect the clinical condition of the dog. However, the arterial hypoxemia mildly improved after the first day of therapy (PaO_2_ increased from 48.5 to 53 mm Hg, reference interval 85–103 mm Hg). The echocardiogram was repeated on the third day of therapy and the echocardiographic changes and the severity of the PH were markedly reduced. The right ventricular dilation had dramatically decreased, the interventricular septum was no longer flattened, and the pressure gradient of the tricuspid regurgitation was reduced from 77 mm Hg to 41 mm Hg (reference < 25 mm Hg) (Fig. 2b). Therefore, therapy with pimobendan and sildenafil was continued during the hospitalized period (7 days).

**Fig. 3:**
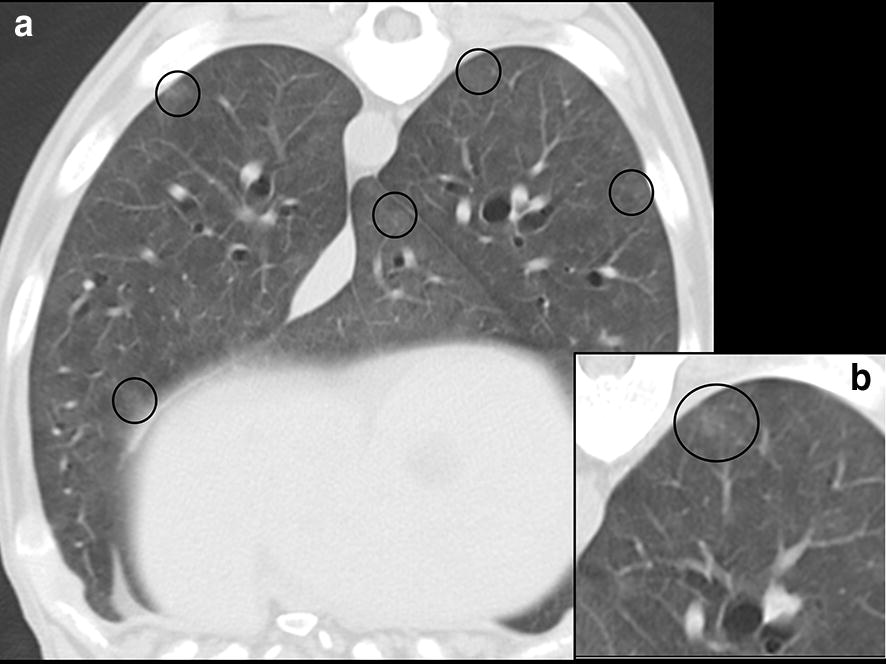
Computed tomography scan images of the lungs. **a** Presence of diffuse, small, poorly circumscribed centrilobular ground glass nodules (circles) throughout the lungs. **b** Close-up of a ground glass nodule (circle)

As further diagnostic steps a (pre-and post-contrast) computed tomography (CT) scan with an apnoea, followed by a surgical lung biopsy in the same anaesthetic session were performed 4 days after the initial presentation. A single slice helical CT scanner (Philips Secura, Philips NV, Eindhoven, the Netherlands) was used. Technical settings included 3 mm helical slices, 120 kV, 200 mA, 292 mm field of view, 512 × 512 matrix and a high spatial frequency algorithm. On CT images, the lung parenchyma showed subtle centrilobular ground glass nodules and an enlarged pulmonary artery. Septal lines, pleural effusion and lymphadenopathy were absent (Fig. 3a, b). CT findings were compatible with PH without a conclusive diagnosis. Following the CT scan, a lung biopsy from the left cranial lung lobe (3 × 2 cm) was taken for histopathological examination with a mini-thoracotomy. Directly after this procedure a therapy with dexamethasone (0.25 mg/kg q24 h I.V., Rapidexon ^®^, Eurovet Animal Health, Bladel, the Netherlands) was initiated as a last resort while histopathological results were pending (3 days).

Histopathology of the surgical lung biopsy showed a moderate chronic interstitial histiocytic pneumonia of unknown aetiology and atelectasis by haematoxylin and eosin, periodic acid–Schiff, and Van Gieson’s stainings. Vascular changes were initially not clearly identified. Because of the poor response to the initiated treatment and the suspected poor prognosis based on the histopathologic results, the dog was euthanized. Autopsy was performed with the owner’s informed consent.

**Fig. 4:**
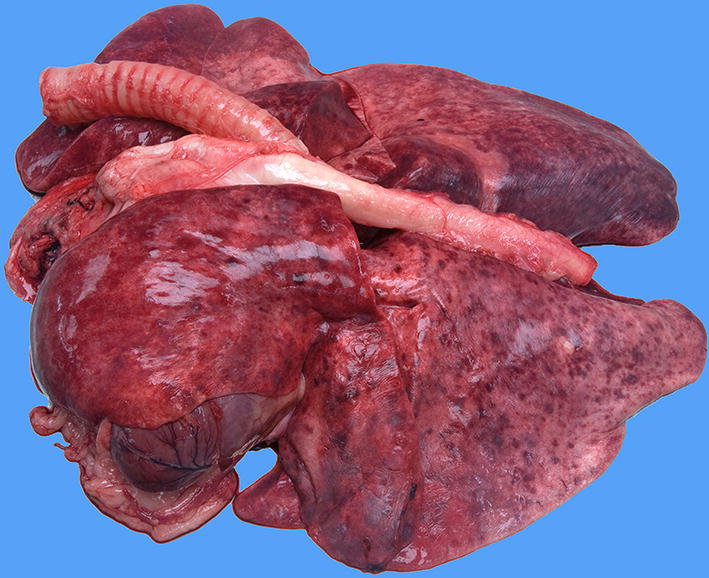
Macroscopic image of the lungs. Macroscopic image of the lungs showing a diffuse mottled appearance with multiple dark red foci of 1 × 1 × 3 mm, and small numbers of white foci of 1 × 1 × 1 mm often surrounded by a dark red zone (demarcation) randomly distributed throughout all lung lobes

**Fig. 5:**
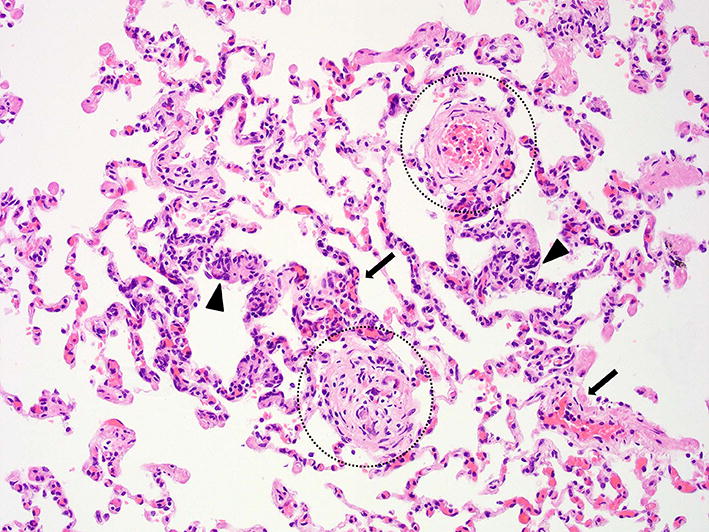
Histologic appearance of the lungs demonstrating remodelling of pulmonary venules, foci of pulmonary capillary hemangiomatosis and segmental alveolar capillary congestion. The upper circle surrounds a relatively normal pulmonary venule, where the lower circle surrounds a vein with severe remodelling (intimal fibrosis and complete obstruction of the lumen), the arrows point to areas with segmental alveolar capillary congestion and the arrowheads indicate foci of pulmonary capillary hemangiomatosis adjacent to the remodelled veins. Haematoxylin and eosin, bar = 50 µm

**Fig. 6:**
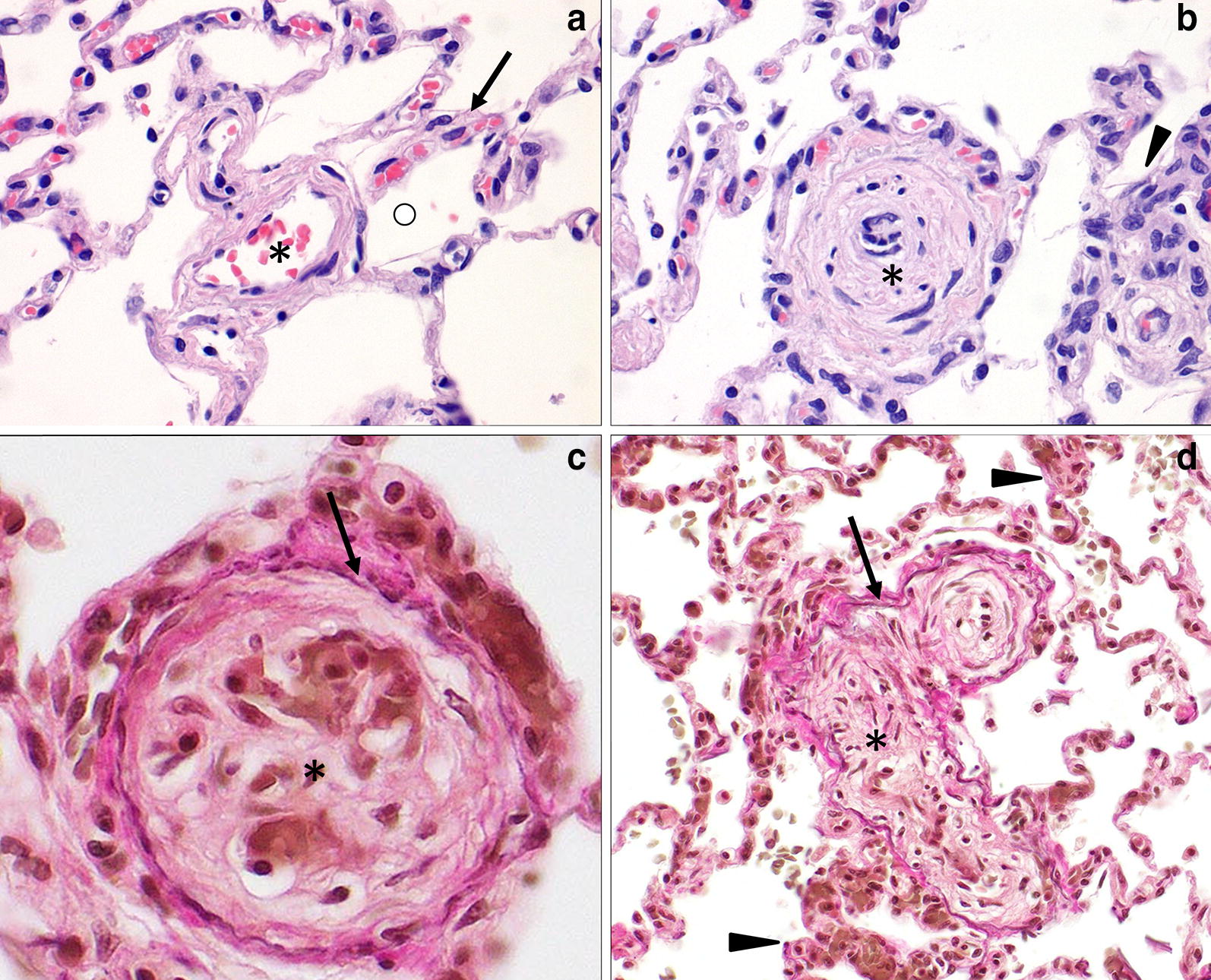
Histologic appearance of the lungs demonstrating remodelling of pulmonary venules. **a** Normal pulmonary architecture. Circle: alveolus, asterisk: lumen of vein, arrow: normal capillary with endothelial cells and erythrocytes. Hematoxylin and eosin. **b** Pulmonary venule with severe intimal fibrosis and obliteration of the lumen (asterisk) with adjacent focus of pulmonary capillary hemangiomatosis (PCH) (arrowhead). Hematoxylin and eosin. **c** Transverse image of a remodeled inter-alveolar pulmonary venule with severe obliteration of the lumen (asterisk) and changes suggestive of post-thrombotic recanalization. The identity of the vessel as a vein was confirmed by the presence of a single external elastic lamina (arrow). Weigert’s resorcine fuchsine. **d** Longitudinal image of an abnormal pulmonary venule with remodeling and severe obliteration of the tortuous lumen (asterisk). The identity of the vessel as a vein was confirmed by the presence of a single external elastic lamina (arrow). The arrowheads indicate foci of PCH. Weigert’s resorcine fuchsine

**Fig. 7:**
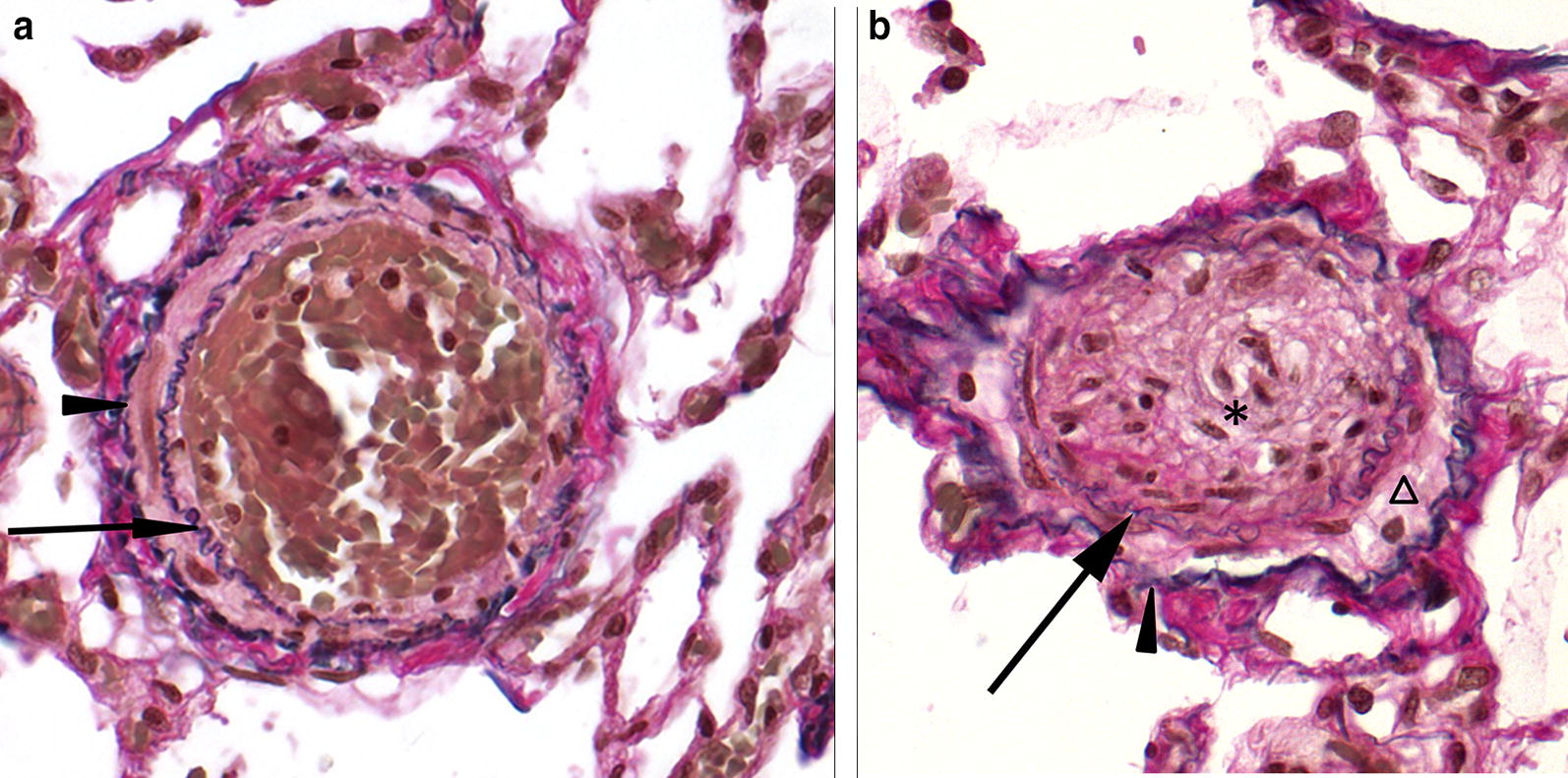
Histologic appearance of the lungs demonstrating remodeling of pulmonary arterioles. **a** Normal pulmonary arteriole demonstrating a lamina elastica interna (*arrow*) and a lamina elastica externa (arrowhead). Weigert’s resorcine fuchsine. **b** Remodeled pulmonary arteriole with concentric intimal proliferation of fibrous tissue causing obliteration of the lumen (asterisk). Medial hypertrophy is also visible (triangle). Arrow indicates the lamina elastica interna, arrowhead indicates lamina elastica externa. Weigert’s resorcine fuchsine

Gross pathology of the lungs showed moderately collapsed firm lungs with a diffuse mottled appearance with multiple dark red foci of 1 × 1 × 3 mm, and small numbers of white foci of 1 × 1 × 1 mm, often surrounded by a dark red zone (demarcation) randomly distributed throughout all lung lobes (Fig. 4). Routine histopathology of the lungs showed multifocal vascular remodelling. In order to differentiate between small arteries and veins, essential to diagnose PVOD, an additional stain visualising elastic fibers and collagen (Weigert’s Resorcin Fuchsin) was added to identify elastic laminae. In this dog, like in people with PVOD, all three compartments (arteries, veins and capillaries) of the pulmonary microcirculation were affected, although the changes in the pulmonary venous system were the most pronounced. Venular lesions included severe concentric intimal proliferation, partial to complete obliteration of the lumina (Figs. 5, 6) and post-thrombotic recanalization (Fig. 6c). Capillary lesions were organized in foci, most obvious adjacent to remodelled venules and were characterized by proliferation of plumb endothelial cells (similar to PCH) (Figs. 5, 6b, d). Segmental congestion of alveolar capillaries was also regularly associated with the foci of PCH (Fig. 5). Arterial lesions resembled those of PAH with concentric intimal thickening by increased extracellular matrix and medial hypertrophy, but complex plexiform lesions were absent (Fig. 7). These findings were consistent with the findings described by Williams et al. [11] and therefore the human equivalent of PVOD.

The genomic DNA of the case was isolated from EDTA-blood using a semi-automated Chemagen extraction robot (PerkinElmer Chemagen Technologie GmbH) and was stored at − 20 °C. Approximately 6 years later, the genomic DNA of the case and an unrelated healthy GSD were analysed by whole genome sequencing. Integrity of DNA was checked on a Bioanalyzer (Agilent, Santa Clara, USA) and quantified using Qubit dsDNA HS (Thermo Fisher Scientific, Waltham, USA). DNA libraries were prepared using TruSeq Nano library prep kit (TruSeq Nano library prep kit, Illumina, San Diego CA, USA) using 200 ng gDNA input. Whole genome sequencing information at 30× coverage was obtained using a HiSeqX Ten instrument (HiSeqX Ten instrument, Illumina, San Diego CA, USA) and 2 × 150 base pair paired-end reads.

Data was processed with our in-house developed pipeline v1.2.1 (https://github.com/CuppenResearch/IAP) including somatic mutation analysis (Strelka, VarScan, FreeBayes, and MuTect) and a genome analysis toolkit (GATK v. 3.2.2) [12] according to best practices guidelines [13]. Sequence reads were mapped against the Canine Reference Genome (CanFam 3.1) using Burrows-Wheeler alignment with maximal exact matches (BWA-MEM) v0.7.5a [14] followed by marked duplicates, merging of lanes, and realignment of indels. Base recalibration was not performed. In human medicine, mutations in *BMPR2, ACVRL1, ENG*, *KCNK3, CAV*-*1*, and *SMAD9* have been suggested to be causative for an autosomal dominant form of PAH [1]. These genes were included in analyses to prevent missing candidate mutations due to phaenotypical misclassification. Comparing the case and control, analysis of these genes revealed 196 unique intronic variants. Comparing the sequence of *EIF2AK4* in the case and the healthy GSD revealed 124 unique variants, of which 9 were located in the 3`-UTR, 112 were intronic and 3 were exonic variants. The case was homozygous mutant for c.2961T>C and c.1266G>A, a heterozygous variation was found at c.2092G>A. Variant validation was performed by Sanger sequencing on amplified polymerase chain reaction products from genomic DNA using Platinum Taq Polymerase (Invitrogen). After exonuclease I treatment DNA sequence reactions were performed using BigDye v3.1 on, sequenced on an ABI3130XL and analyzed in Lasergene (version 12.0 DNASTAR). Four of the five additional GSD's, unknown for any form of respiratory stress and aged ≥ 10 years revealed the identical genotype as the case and therefore excluded these as causal variants for PVOD.

## Discussion and conclusions

The dog in this report was presented with acute onset dyspnea which was also the most common symptom in the 11 dogs with PVOD that were presented in the study of Williams et al. [11]. In humans, respiratory distress is also one of the main clinical symptoms, but the development of it is generally more gradual. Acute decline is however rarely described following episodes of haemoptysis [1]. PVOD can affect humans of all ages, with some reports suggesting a higher frequency in children [5, 15]. The dog described in this study was 11-year old resembling the reported median age of 10.5 years [11]. The reasons for the differences in clinical presentation between humans and dogs are currently unclear and further studies are needed. Nevertheless, besides these differences, many other clinical findings in dogs seem to be similar to those in humans with PVOD. In humans with idiopathic or heritable PAH, arterial oxygen pressure (PaO_2_) remains normal or is only slightly decreased at rest. In contrast, humans diagnosed with PVOD typically demonstrate major resting hypoxemia [1], as was also noted in the dog presented in this case.

Due to the nonspecific findings, plain radiography is of limited value for diagnosing POVD in humans and dogs. This dog only showed pulmonary arterial enlargement on plain thoracic radiographs. In people, high-resolution CT scan has a role in the non-invasive diagnosis of PVOD. Of the histologically proven PVOD patients, 75% demonstrate 2 of the 3 following main characteristic findings on CT: (1) centrilobular ground-glass opacities (i.e., areas of increased attenuation with preserved bronchial and vascular markings located in the central portion of the secondary pulmonary lobule), (2) mediastinal lymph node enlargement and (3) septal lines (i.e., thin lines that can be seen when the interlobular septa in the pulmonary interstitium become prominent) [1]. These findings can aid the diagnosis if the clinical suspicion is high [1] but are in general unspecific and often related to pulmonary oedema or infections.

In this case, CT images showed subtle centrilobular ground glass nodules in the lung parenchyma, but mediastinal lymph node enlargement was not found. Septal lines were also not visible, but these were not expected to occur, since dogs do not have interlobular septa.

Histopathology of the lung specimens obtained *post*-*mortem* revealed the presence of vascular changes that were deemed consistent with PVOD after consultation of a human pathologist. Although these vascular changes were initially not recognized on histopathology of the surgical lung biopsy, re-evaluation of the biopsied tissue did show similar vascular pathology. However, in some cases a single histological lung biopsy may not be sufficient to diagnose PVOD, because venous remodelling is not always evenly distributed and normal veins can still be found in certain lung regions. Therefore, multiple biopsies from different locations are recommended in suspect cases of PVOD.

Genetic testing for *EIF2AK4* mutations is advised for humans with sporadic or familial PVOD. The presence of a bi-allelic *EIF2AK4* mutation is sufficient to confirm a diagnosis of PVOD without performing a hazardous lung biopsy for histological confirmation [1]. In this dog, no causal variants were found in *EIF2AK4* gene, nor within the human PAH associated genes.

Treatment of PH is aimed at eliminating or improving the underlying disease process and is used to control clinical symptoms like syncope and right-sided congestive heart-failure. If the PH is either idiopathic or does not improve by primary disease therapy, treatment with vasodilators may be implemented. However, in contrast to patients with PAH, treatment with vasodilators can result in life-threatening pulmonary oedema in patients with PVOD [5]. A speculative explanation for this complication is that PAH-specific vasodilators might cause an increase in hydrostatic pressure, due to an augmented pulmonary arterial blood flow against the fixed resistance of the occluded venules. This can cause vascular leakage, which can progress to severe pulmonary oedema. However, some success has been shown in treatment of carefully selected people with PVOD with prostacyclin, bosentan, and/or sildenafil in small case series [16, 17]. In the present case, a dual therapy with pulmonary arterial dilators (sildenafil and pimobendan) was initiated, because we hoped this would ameliorate the very severe PH and clinical signs faster than a monotherapy with sildenafil. We fortunately did not notice a decline in the clinical condition or an increase of the hypoxaemia after the initiation of the phosphodiesterase inhibitors. Thoracic radiographs were not repeated, but no evidence of pulmonary oedema was found on the CT images 4 days after initiation of the vasodilator therapy. In humans, no evidence-based medical therapy exists for PVOD at present, and lung transplantation remains the preferred definitive therapy for eligible patients [1].

In conclusion, this case report confirms that PVOD should be a diagnostic consideration in dogs presenting with dyspnea and pulmonary hypertension of unknown aetiology. In this dog, no causal variants were found in the genes that are known to be involved in humans with PVOD and PAH. More studies are necessary to better describe the presentation of canine PVOD which will hopefully aid in the prospective identification of cases and clinical management of canine PVOD.

## Abbreviations

ABG: arterial blood gas analysis
CT: computed tomography
*EIF2AK4*: eukaryotic translation initiation factor 2 alpha kinase
GSD: German Shepherd dog
PAH: pulmonary arterial hypertension
PCH: pulmonary capillary hemangiomatosis
PH: pulmonary hypertension
PVOD: pulmonary veno-occlusive disease
